# Supplementary material for: Quantitative live imaging reveals PRICKLE1 controls junctional neural tube morphogenesis independent of Planar Cell Polarity
Source: Nat Commun. 2026 Apr 27;17:3654. doi: 10.1038/s41467-026-71242-0 (PMC13121753; doi:10.1038/s41467-026-71242-0)
Supplement: Supplementary file 1 — Supplementary Information [file 41467_2026_71242_MOESM1_ESM.pdf]

SUPPLEMENTARY FIGURES

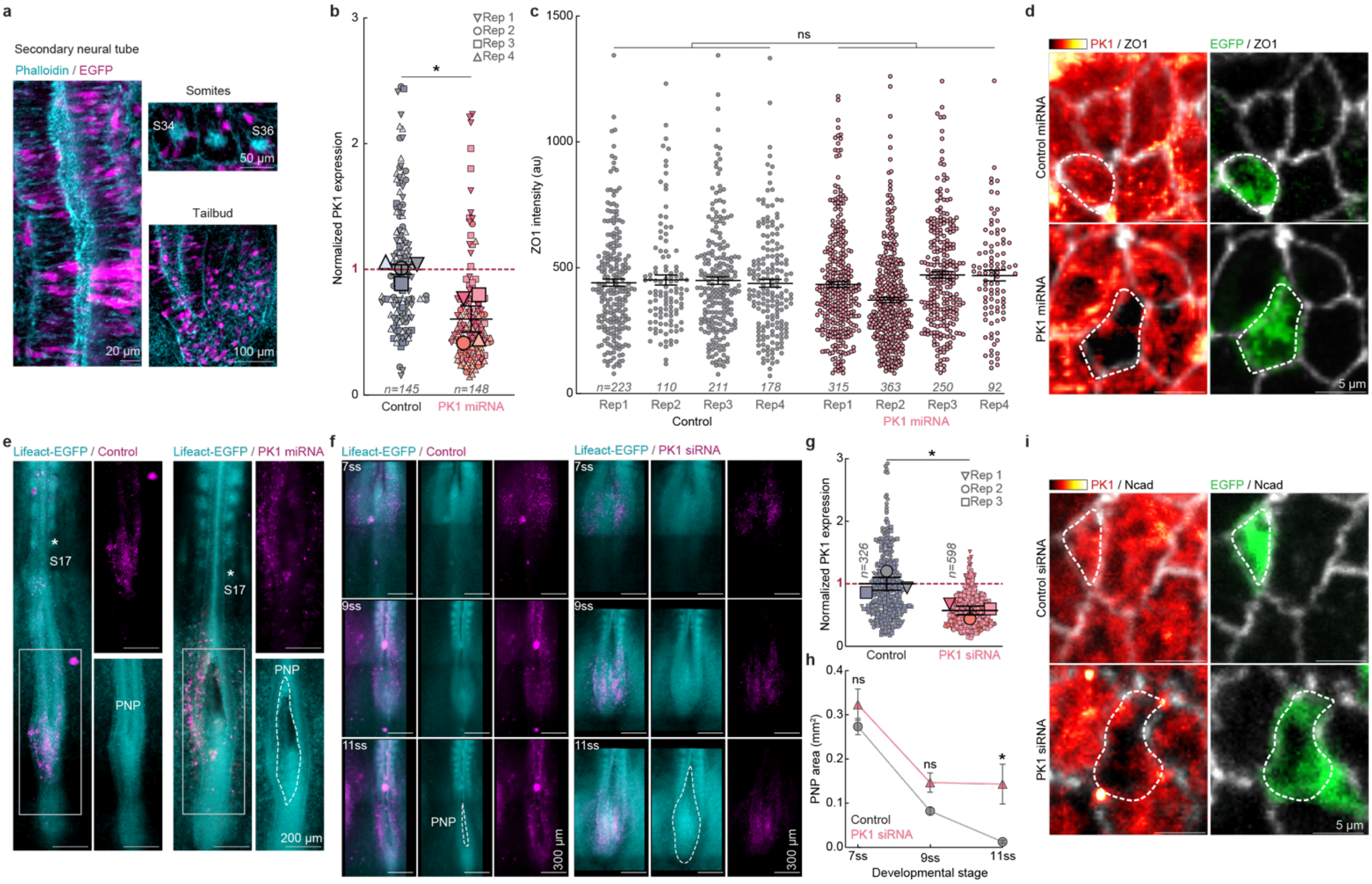

Supplementary Fig. 1. PK1 knockdown causes sustained disruption of neural tube closure, related to Fig. 1

a Cells electroporated in the junctional zone at the 6ss contribute to the secondary neural tube, sparsely to somites 34 to 36 and to the tailbud. **b** PK1-knockdown miRNA decreases the endogenous PK1 expression level in the JZ. The expression level of PK1 is normalized by that of ZO1, mean, ± sem, (n = 145 cells from 4 embryos for Control and n = 148 cells from 4 embryos for PK1 miRNA), p = 0.0114, two-sided un-paired t-test. **c** PK1 knockdown does not affect the ZO1 expression level, mean, ± sem, (n = 722 cells from 4 embryos for Control and n = 1020 cells from 4 embryos for PK1 miRNA), p = 0.7361, ns, not significant, two-sided un-paired t-test. **d** PK1 expression is reduced by the PK1 miRNA but not the scrambled control miRNA. **e** PK1 knockdown results in sustained defects in posterior neuropore (PNP) closure at 17ss. **f - i** PK1 knockdown by siRNA generates comparable defects to the PK1 miRNA, mean, ± sem, .For g, (n = 326 cells from 3 control embryos and n = 598 cells from 3 PK1 KD embryos), p = 0.0269, for h, (n = 3 embryos for both control and KD group), p = 0.2780, 0.0739 and 0.0445 for 7ss to 9ss, ns, not significant, two-sided un-paired t-test.

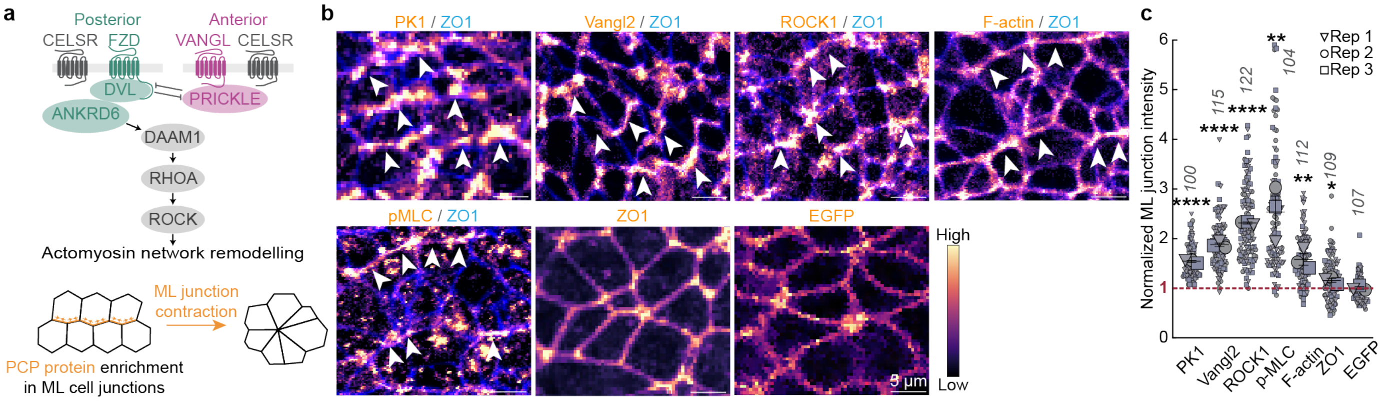

Supplementary Fig. 2. PCP signalling components are polarized along mediolateral cell junctions, related to Fig. 2

a Schematic of Planar Cell Polarity signalling for actomyosin remodeling and mediolateral (ML) cell junction contraction. **b, c** PCP components are preferentially enriched along the mediolateral (ML) cell junctions. The polarity of PCP proteins is compared to that of the membrane EGFP from transgenic quail embryos, which exhibits no apparent subcellular polarisation, mean, ± sem (n = 100 cells from 3 embryos for PK1, n = 115 cells from 3 embryos for Vangl2, n = 122 cells from 3 embryos for ROCK1, n = 104 cells from 3 embryos for p-MLC, n = 112 cells from 3 embryos for F-actin, n = 109 cells from 3 embryos for ZO1, n = 107 cells from 3 embryos for EGFP), p = 9.0×10^{-5}, 4.7×10^{-5}, 3.1×10^{-6}, 0.0088, 0.0084 and 0.0327 for PK1, Vangl2, ROCK1, p-MLC, F-actin and ZO1, two-sided un-paired t-test.

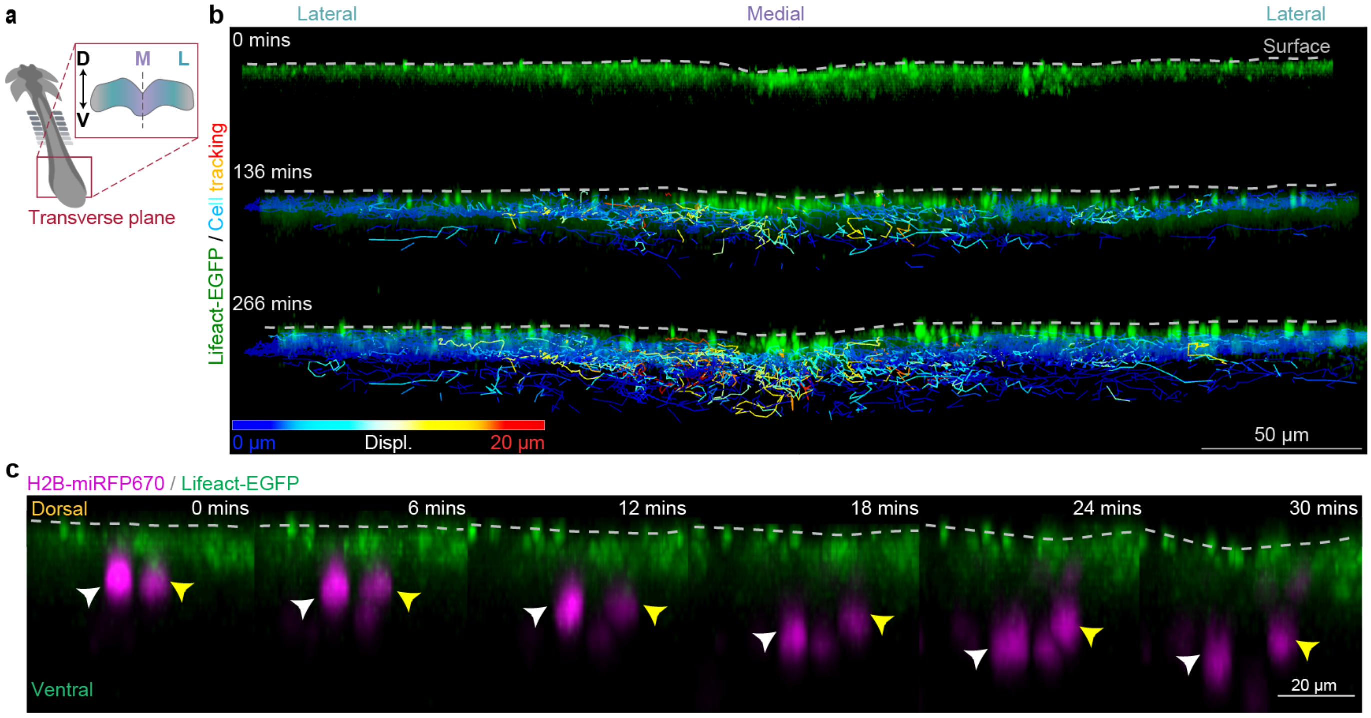

Supplementary Fig. 3. Ingressing cells move ventrally relative to the dorsal surface of the tissue, related to Fig. 3

a Schematic showing region of interest. **b** Snapshot showing tracks of H2B-miRFP670 labelled cells migrating along the dorsoventral axis of the JZ in a Lifeact-EGFP embryo. **c** Timelapse showing individual H2B-miRFP670 labelled cells (arrowheads) moving ventrally inside the embryo over 30 minutes.

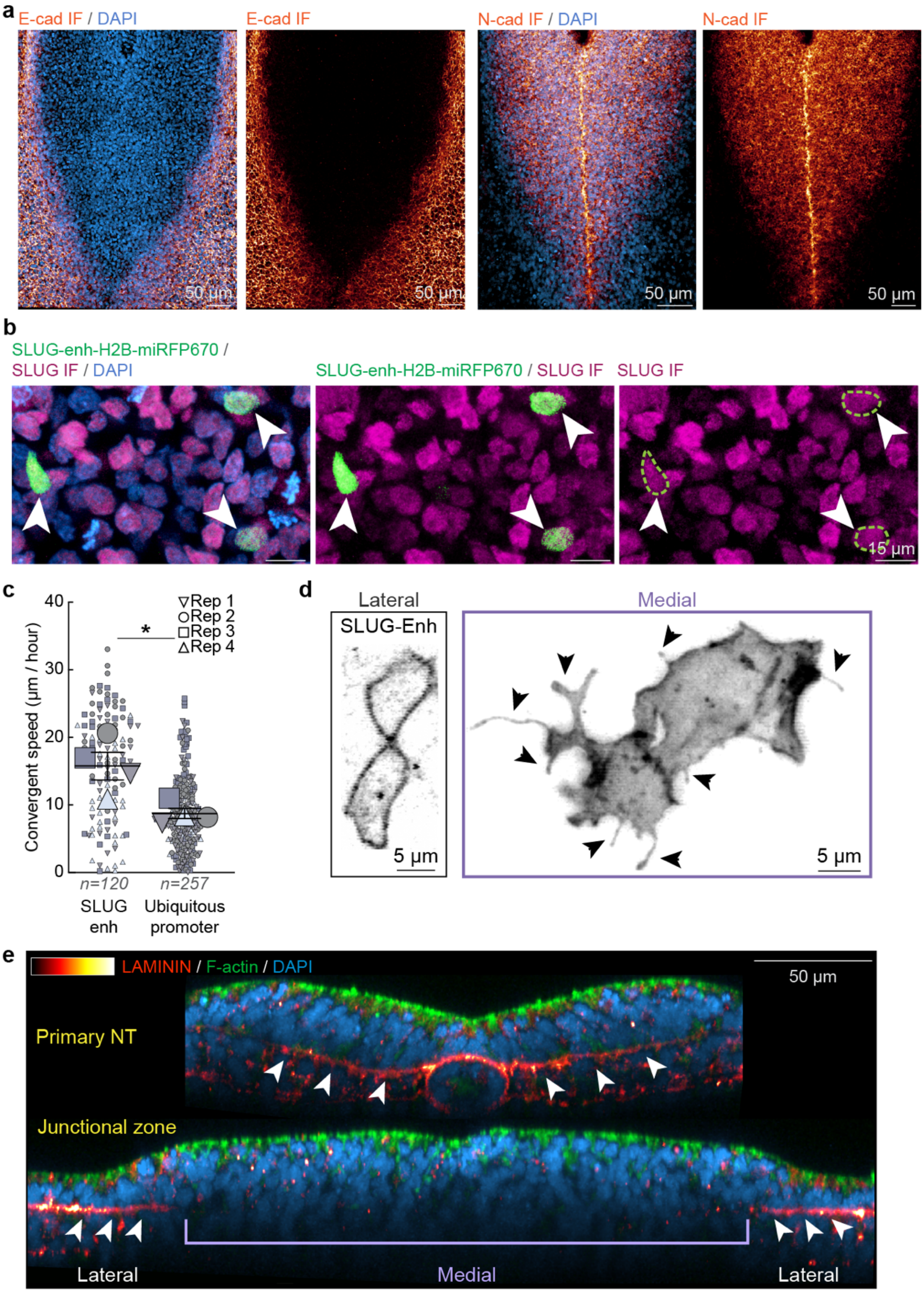

Supplementary Fig. 4. SLUG⁺ cells undergo a partial EMT, related to Fig. 4

a Cells in the JZ express N-cadherin and not E-cadherin by the 6ss**. b** Cells expressing H2B-miRFP670 under the control of the SLUG enhancer (SLUG enh-H2B-miRFP670) are also positive for SLUG immunofluorescent staining. **c** SLUG enhancer-positive cells, mean, ± sem (n = 120 cells from 4 embryos) converge significantly faster than cells labelled with ubiquitous CMV promoter (n = 257 cells from 4 embryos), p = 0.0183, two-sided un-paired t-test. **d** SLUG enhancer-positive cells have an epithelial morphology in the lateral JZ but a protrusive mesenchymal morphology (arrowheads) in the medial JZ. **e** A basement membrane rich in LAMININ (arrowheads) sits underneath the primary neural tube but is absent from the medial region of the JZ.

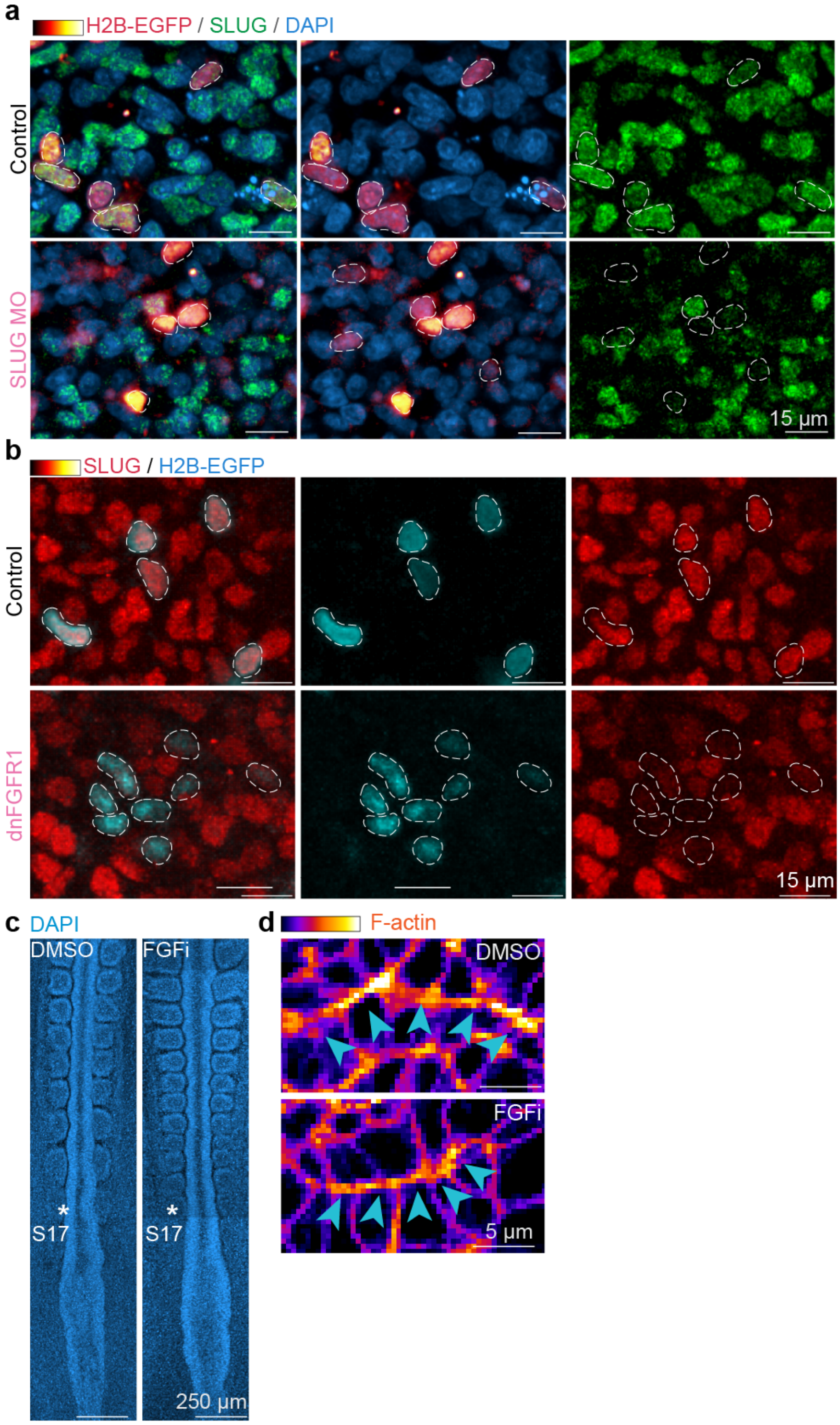

Supplementary Fig. 5. FGF signalling regulates SLUG expression in the junctional zone but is not required for supracellular actin cable formation, related to Fig. 5

a Electroporation of a SLUG morpholino (MO) effectively reduces the number of SLUG+ cells in the JZ. The morpholino is co-electroporated with H2B-EGFP (in red). **b** Electroporation of a dnFGFR1 construct reduces the expression of SLUG (red) in the JZ. **c** Embryos treated with FGF inhibitor after the closure of the posterior neuropore appear morphologically normal. **d** FGF-inhibited embryos still assemble F-actin supracellular cables (arrowheads) in the lateral JZ.

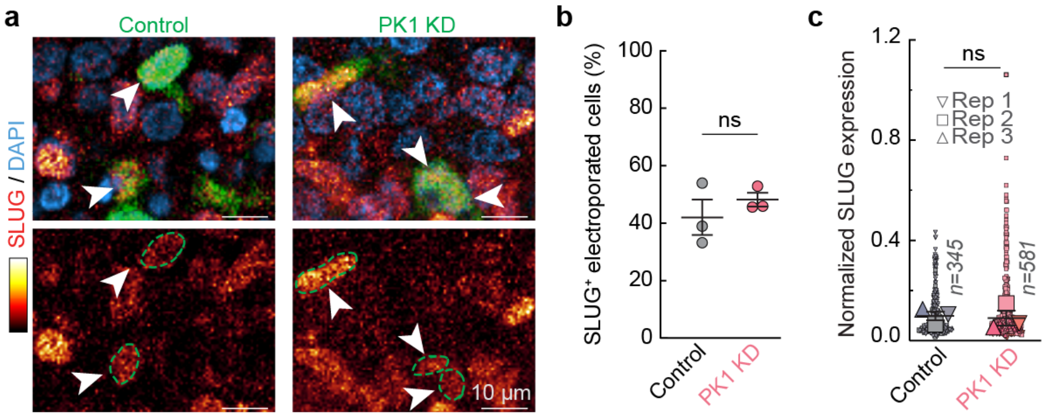

Supplementary Fig. 6. PK1 knockdown does not alter SLUG expression, related to Fig. 6

a Immunofluorescent staining for SLUG reveals that Control and PK1 knockdown cells (green) still express SLUG (white arrowheads). **b, c** Quantification shows no change in the percentage of SLUG$^+$ cells or levels of SLUG expression following PK1 knockdown compared with Control, mean, ± sem (n = 345 cells from 3 Control embryos and n = 581 cells from 3 PK1 KD embryos), p = 0.406 for b and 0.8307 for c, ns, not significant, two-sided un-paired t-test.

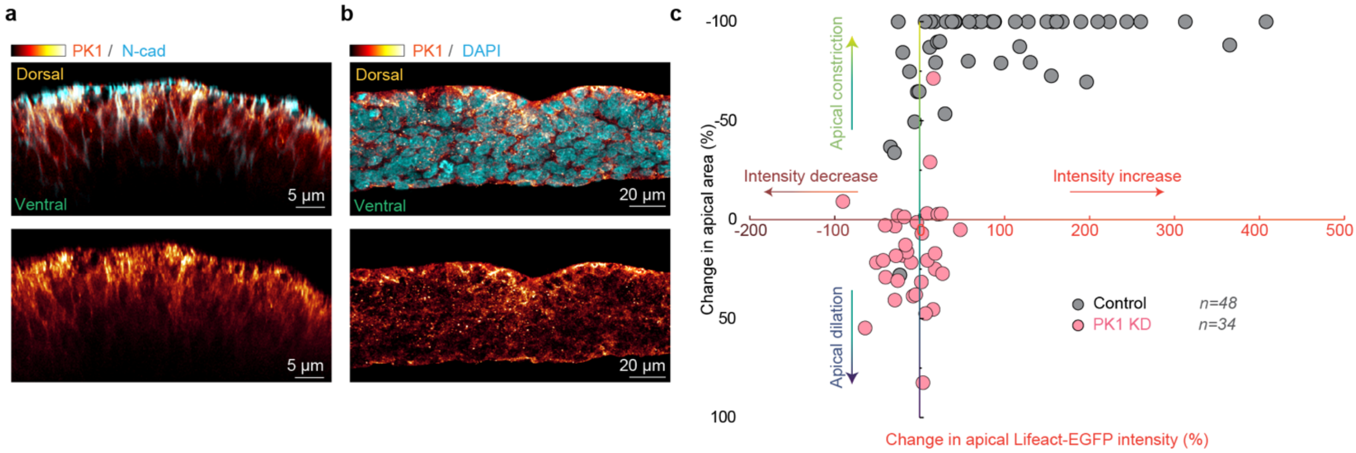

Supplementary Fig. 7. PK1 is apically localized and knockdown inhibits both the cortical F-actin enrichment and apical constriction, related to Fig. 7

a X, Z view of a wholemount quail embryo immunostained for N-cadherin and PK1 shows apical localisation of both. **b** Immunostaining of a transverse vibratome section through the quail JZ shows PK1 expression on the apical side. **c** Live imaging of electroporated Lifeact-EGFP quail embryos reveals increased Lifeact-EGFP intensity at the apical cortex of Scrambled control cells as their apical surface constricts (n = 48 cells from 3 Control embryos and n = 34 cells from 3 PK1 KD embryos). PK1 knockdown cells fail to increase Lifeact-EGFP at their apical surface and do not constrict.
