# Supplementary material for: Quantitative live imaging reveals PRICKLE1 controls junctional neural tube morphogenesis independent of Planar Cell Polarity
Source: Nat Commun. 2026 Apr 27;17:3654. doi: 10.1038/s41467-026-71242-0 (PMC13121753; doi:10.1038/s41467-026-71242-0)
Supplement: Supplementary file 2 — Description of Additional Supplementary Files [file 41467_2026_71242_MOESM2_ESM.pdf]

Description of Additional Supplementary Files

SUPPLEMENTARY MOVIES

Supplementary Movie 1. JZ converges without extension, related to Fig. 1

Supplementary Movie 2. Cells in JZ converge, related to Fig. 1

Supplementary Movie 3. PK1 knockdown abolishes PNP closure, related to Fig. 1

Supplementary Movie 4. PK1 knockdown disrupts cellular convergence, related to Fig. 2

Supplementary Movie 5. Cells in medial JZ ingress, related to Fig. 3

Supplementary Movie 6. PK1 knockdown impairs medial cellular ingression, related to Fig. 3

Supplementary Movie 7. SLUG positive cells migrate medially and ingress, related to Fig. 4

Supplementary Movie 8. PK1 knockdown perturbs cell apical constriction and ingression, related to Fig. 6
