# Supplementary material for: Quantitative live imaging reveals PRICKLE1 controls junctional neural tube morphogenesis independent of Planar Cell Polarity
Source: Nat Commun. 2026 Apr 27;17:3654. doi: 10.1038/s41467-026-71242-0 (PMC13121753; doi:10.1038/s41467-026-71242-0)
Supplement: Supplementary file 11 — Reporting Summary [file 41467_2026_71242_MOESM11_ESM.pdf]

nature portfolio

Corresponding author(s): White
Last updated by author(s): Feb 25, 2026

Reporting Summary

Nature Portfolio wishes to improve the reproducibility of the work that we publish. This form provides structure for consistency and transparency in reporting. For further information on Nature Portfolio policies, see our Editorial Policies and the Editorial Policy Checklist.

Statistics

For all statistical analyses, confirm that the following items are present in the figure legend, table legend, main text, or Methods section.

n/a	Confirmed	
☐	☒	The exact sample size (*n*) for each experimental group/condition, given as a discrete number and unit of measurement
☐	☒	A statement on whether measurements were taken from distinct samples or whether the same sample was measured repeatedly
☐	☒	The statistical test(s) used AND whether they are one- or two-sided<br>*Only common tests should be described solely by name; describe more complex techniques in the Methods section.*
☒	☐	A description of all covariates tested
☐	☒	A description of any assumptions or corrections, such as tests of normality and adjustment for multiple comparisons
☐	☒	A full description of the statistical parameters including central tendency (e.g. means) or other basic estimates (e.g. regression coefficient) AND variation (e.g. standard deviation) or associated estimates of uncertainty (e.g. confidence intervals)
☐	☒	For null hypothesis testing, the test statistic (e.g. *F*, *t*, *r*) with confidence intervals, effect sizes, degrees of freedom and *P* value noted<br>*Give P values as exact values whenever suitable.*
☒	☐	For Bayesian analysis, information on the choice of priors and Markov chain Monte Carlo settings
☒	☐	For hierarchical and complex designs, identification of the appropriate level for tests and full reporting of outcomes
☒	☐	Estimates of effect sizes (e.g. Cohen's *d*, Pearson's *r*), indicating how they were calculated

Our web collection on statistics for biologists contains articles on many of the points above.

Software and code

Policy information about availability of computer code

Data collection	Imaging data was collected using Zeiss Zen Black 2012 and Zeiss Zen Blue 3 (with Guided Acquisition and Experiment Designer)
Data analysis	Data analysis was performed in Fiji 2.14.0, MATLAB R2021a, Graphpad Prism 10.2.0, Snapgene6.2.1 and Microsoft Excel 16.101

For manuscripts utilizing custom algorithms or software that are central to the research but not yet described in published literature, software must be made available to editors and reviewers. We strongly encourage code deposition in a community repository (e.g. GitHub). See the Nature Portfolio guidelines for submitting code & software for further information.

Data

Policy information about availability of data

All manuscripts must include a data availability statement. This statement should provide the following information, where applicable:

- Accession codes, unique identifiers, or web links for publicly available datasets
- A description of any restrictions on data availability
- For clinical datasets or third party data, please ensure that the statement adheres to our policy

Source data are provided with this study. The raw imaging data and codes underlying the figures in this study have been deposited at The University of Queensland's institutional repository, UQ eSpace, and are publicly available at: https://doi.org/10.48610/5c66443.

Research involving human participants, their data, or biological material

Policy information about studies with human participants or human data. See also policy information about sex, gender (identity/presentation), and sexual orientation and race, ethnicity and racism.

Reporting on sex and gender	n/a
Reporting on race, ethnicity, or other socially relevant groupings	n/a
Population characteristics	n/a
Recruitment	n/a
Ethics oversight	n/a

Note that full information on the approval of the study protocol must also be provided in the manuscript.

Field-specific reporting

Please select the one below that is the best fit for your research. If you are not sure, read the appropriate sections before making your selection.

☒ Life sciences ☐ Behavioural & social sciences ☐ Ecological, evolutionary & environmental sciences

For a reference copy of the document with all sections, see nature.com/documents/nr-reporting-summary-flat.pdf

Life sciences study design

All studies must disclose on these points even when the disclosure is negative.

Sample size	No statistical methods were used to determine sample size. Sample size was chosen based on prior experience and standards in the field.
Data exclusions	No data was excluded
Replication	All experiments were performed as technical and biological replicates (n = minimum of 3) with similar outcome.
Randomization	Embryos were allocated to experimental and control groups in an alternating manner as they were prepared. Control and experimental conditions were distributed across multiple clutches and experimental days to minimize batch effects.
Blinding	Investigators were not blinded to experimental condition during imaging, but quantitative analyses were performed using automated or predefined criteria.

Reporting for specific materials, systems and methods

We require information from authors about some types of materials, experimental systems and methods used in many studies. Here, indicate whether each material, system or method listed is relevant to your study. If you are not sure if a list item applies to your research, read the appropriate section before selecting a response.

Materials & experimental systems

n/a	Involved in the study	
☐	☒	Antibodies
☒	☐	Eukaryotic cell lines
☒	☐	Palaeontology and archaeology
☐	☒	Animals and other organisms
☒	☐	Clinical data
☒	☐	Dual use research of concern
☒	☐	Plants

Methods

n/a	Involved in the study	
☒	☐	ChIP-seq
☒	☐	Flow cytometry
☒	☐	MRI-based neuroimaging

Antibodies

Antibodies used	SOX2 (Abcam, AB97959, 1:200), PRICKLE1 (Proteintech, 22589-1-AP, 1:200), ZO-1 (Thermo Fisher Scientific, 33-9100 and 40-2200, 1;400), VANGL2 (Sigma Aldrich, MABN750, 1:50), ROCK1 (ABClonal, A11158, 1:100), p-MLC (ABClonal, AP1433, 1:100), Fibronectin

	(DSHB, B3/D6-s, 1:4), Fibronectin (Sigma Aldrich, F3648, 1:100), SLUG (New England Biolabs, 9585T, 1:100), N-Cadherin (DSHB, 6B3-s, 1:100), E-Cadherin (BD Biosciences, 610181, 1:100).
Validation	SOX2 (PMID: 21750029), PRICKLE1 (PMID: 38727576), ZO-1 (PMID: 38727576 for 33-9100 and PMID: 35805087 for 40-2200), VANGL2 (PMID: 35580169), ROCK1 (PMID: 39430840), p-MLC (PMID: 38309866), Fibronectin (PMID: 21412939 for DSHB), Fibronectin (PMID: 34472370 for sigma), SLUG (PMID: 29084958), N-Cadherin (PMID: 30459275), E-Cadherin (PMID: 24297751).

Animals and other research organisms

Policy information about studies involving animals; ARRIVE guidelines recommended for reporting animal research, and Sex and Gender in Research

Laboratory animals	Japonica coturnix aged between 8 weeks - 2 years were used to provide fertilised eggs for experiments.
Wild animals	n/a
Reporting on sex	Sex was not determined for quail embryos used in this study, as embryos were analyzed at early developmental stages prior to sexual differentiation. Sex was therefore not considered as a biological variable.
Field-collected samples	n/a
Ethics oversight	Ethics approval was provided by the University of Queensland Animal Ethics Committee for the breeding of quails to provide fertilised eggs.

Note that full information on the approval of the study protocol must also be provided in the manuscript.

Plants

Seed stocks	n/a
Novel plant genotypes	n/a
Authentication	n/a
